# The impact of the ultrasonic, bipolar and integrated energy devices in the adrenal gland surgery: literature review and our experience

**DOI:** 10.1186/s12893-018-0457-5

**Published:** 2019-04-24

**Authors:** Renato Patrone, Claudio Gambardella, Roberto Maria Romano, Clarizia Gugliemo, Chiara Offi, Claudia Andretta, Antonio Vitiello, Ernesto Tartaglia, Luigi Flagiello, Alessandra Conzo, Claudio Mauriello, Giovanni Conzo

**Affiliations:** 1 Division of General and Oncologic Surgery - Department of Cardiothoracic Sciences, University of Campania “Luigi Vanvitelli”, Via Pansini 1, 80131 Naples, Italy; 2 Department of Surgery and Clinic Medicine, Federico II University of Naples, via pansini 1, 80131 Naples, Italy; 3 Department of General, Laparoscopic and Robotic Surgery, Azienda Ospedaliera, Specialistica Dei Colli - Monaldi Hospital, Via Leonardo Bianchi, 80131 Naples, Italy; 4 Department of Anesthesiologic, Surgical and Emergency Sciences Second University of Naples- Italy, Via Sergio Pansini 5, 80131 Naples, Italy

**Keywords:** Laparoscopic adrenalectomy, Hemostatic devices, Minimally invasive adrenalectomy, Harmonic Focus, Ligasure, Thunderbeat

## Abstract

**Background:**

The gold standard approach for surgical treatment of benign and malignant adrenal lesion is considered the laparoscopic one, due to a lot of advantages compared to open approach. The rapid propagation of this surgical technique is due to the diffusion of haemostatic devices in laparoscopic adrenal surgery.

The principal aim of this study is to analyze the outcome of LA using each energy modality, evaluating the eventual superiority of an instrument over the others.

**Methods:**

A retrospective study, involving 75 consecutive patients submitted to LA by transperitoneal lateral approach from January 2013 to June 2017, was performed. Age less than 70 years old, adrenal adenomas less than 8 cm in diameter, incidentalomas < 6 cm, myelolipomas < 13 cm, adrenal metastases < 7 cm and ASA score ≤ III were the main surgical inclusion criteria. All involved patients were divided into three group, one for each energy device: group 1 - Harmonic Scalpel, group 2 - Ligasure vessel sealing system and group 3 - Thunderbeat. In each group only one device was applied for dissection and haemostasis during the whole operation. Each group consisted of 25 patients, well matched for histology, tumor size and site, gender and age. The following parameters were collected: age, gender, size of the tumor, side of the affected gland, pathology, operating time, intraoperative blood losses, hospitalization time, complication and conversion rate.

**Results:**

There was no significant statistical difference between groups regarding the relationship between male/female, right site/left site, the mean age, hospitalization time and the tumor size (*p* > 0.05). Significant statistical difference are detectable in operation time and intraoperative blood losses. Thunderbeat, compared respectively with Ligasure and Harmonic Scalpel, is the fastest device (*p* < 0,001). The second faster device resulted Harmonic Scalpel, which meanly reduced the operation time compared to Ligasure (*p* = 0.048). intraoperative blood losses are reduced using Thunderbeat (*p* < 0,001) and HS (*p* = 0.006) compared to Ligasure, but between Thunderbeat and Harmonic Scalpel there isn’t significant statistical difference (*p* = 0.178).

**Conclusions:**

Analyzing the results, laparoscopic adrenalectomy carried out using Thunderbeat appeared to show a statistically significant decrease in operation time and intraoperative blood losses compared with laparoscopic adrenalectomy performed using Harmonic Scalpel and Ligasure, while hospitalization time was superimposable in all groups. According to our data, a responsible use of advanced energy devices can improve surgical outcomes guarantying a cost savings and patient’s satisfaction.

## Background

Laparoscopic adrenalectomy (LA) by transabdominal lateral approach is internationally accepted like the gold standard surgical technique to treat adrenal mass, functioning and non-functioning [1, 2]. Compared to open approach, the most important advantages of this technique are the short hospitalization, the reduced morbidity and the improved cosmetics results with a rapid recovery and an increasing patients satisfaction [3]. Nevertheless open surgery is advisable for large malignant adrenocortical tumors and in patient with haemodynamic instability [4].

indication to LA for lesions > 6 cm is still a matter of debate and experienced endocrine surgeons are divided between supporters [5–7] and detractors [8].

The large widespread of LA is directly consequent to the large diffusion and improvement of energy-based devices. Before its routine application, dissection and hemostasis of adrenal-feeding vessels are performed by electric hook and by titanium clips or laparoscopic staplers. Harmonic Scalpel (HS) (Ethicon Endosurgery, Inc., Cincinnati, OH), Ligasure vessel sealing system (LS) (Tyco Valleylab, Boulder, 20,052 Monza, Italy CO) and Thunderbeat (TB) (Olympus Europa Se & Co, Hamburg, Germany) are advanced haemostasis devices, able to seal and cut vessels with the application of different modality of energy (radiofrequency, ultrasound or both).

These devices have wide applications in various surgical fields, laparoscopic or not. In Literature, several Authors have reported studies in which compared the surgical outcomes of LS, HS and TB in thyroid, spleen and liver surgery [9–13]. To date, only two papers compared HS and LS in adrenal surgery, nevertheless in none is analysed the use of all these three devices [14, 15].

The principal aim of this study is to analyze the outcome of LA using each energy modality, evaluating the eventual superiority of an instrument over the others.

## Methods

### Study design

A retrospective study, involving 75 consecutive patients submitted to LA by transperitoneal lateral approach from January 2013 to June 2017, was performed.

All patients were subjected to clinical examination, laboratory exams, chest X-ray, ECG examination and ultrasonography followed by Computed Tomography or Magnetic Resonance Imaging. In case of suspicion of pheochromocytoma (PCC) a 131-I-metaiodobenzylguanedine scintigraphy and RET proto-oncogene mutations genetic study was added [16, 17].

All patients received antithrombotic prophylaxis (sodium heparin 4000 U.I. s.c.) and a single intra-operative antibiotic prophylaxis. Doxazosin 2 mg, an alpha-1-blocker has been administered to PCC patients to achieve blood pressure (BP), heart rate (HR) and ECG stabilization. The goal is considered a BP < 160/90 mmHg and HR < 100 beats min for at least 24 h before surgery, ECG ST normalization for at least one week before surgery. Atenolol 50 mg, a beta-blocker, was administered in case of persistent tachycardia. Any PCC patient needed the use of crystalloid solution preoperatively for a plasma volume expansion.

Potassium Aspartate 3 mEq/ml i.v. and Spironolactone 50 mg has been administered preoperatively in low potassium serum level (Conn’s disease).

Age less than 70 years old, adrenal adenomas less than 8 cm in diameter, incidentalomas < 6 cm, myelolipomas < 13 cm, adrenal metastases < 7 cm and ASA score ≤ III were the main surgical inclusion criteria. Patients with suspicious of primary malignant adrenal neoplasm were not included in the study. Surgical procedures were all performed by an experienced endocrine surgery team.

LA were performed using the standardize surgical technique with transperitoneal lateral laparoscopic approach regardless of used devices. Dissection was realized using HS, LS or TB.

All involved patients were divided into three group, one for each energy device: group 1 - HS (G1), group 2 - LS (G2) and group 3 - TB (G3). In each group only one device was applied for dissection and haemostasis during the whole operation.

Each group consisted of 25 patients, well matched for histology, tumor size and site, gender and age. [Table 1].

**Table 1:** Demographics and pathologic findings

	Group 1 (HS)	Group 2 (LS)	Group 3 (TB)
Number	25	25	25
Age	45.7 (31/80)	49.4 (23/78)	49.5 (33/68)
Gender	18 F/ 7 M	16 F/ 9 M	18 F/ 7 M
OT	95.2′ (75/110)	99.8′ (82/112)	82.7′ (68/120)
IBL	97.4 ml (50/200)	124.9 ml (45/270)	87.8 ml (55/180)
HT	5.1 day (2/10)	4.7 day (3/9)	4.6 day (2/7)
Side	5.2 cm (1.1/11.2)	5.8 cm (3.2/10.5)	6.2 (4.2/12.1)
Site	14 L/11 R	12 L/ 13 R	11 L/ 14 R
CR	0%	0%	0%

*OT* Operating time, *IBL* intraoperative blood losses, *HT* hospitalization time, *CR* conversion rate, *cm* centimeters, *(‘)* minutes. Age, OT, IBL, HT and side are expressed like mean with extreme value

The following parameters were collected: age, gender, size of the tumor, side of the affected gland, pathology, operating time (OT), intraoperative blood losses (IBL), hospitalization time (HT), complication and conversion rate (CR).

In this retrospective sequential study data after 30-day surgery were not collected.

### Energy devices

HS is an ultrasonic surgical shears, able to cut and coagulate using lower temperatures than those used by conventional electrosurgical equipment. The design of these hand-activated shears, reproduces the familiar “Kelly clamp” shape, with very thin and delicate tips. The tool allows the surgeon to easily dissect, besides coagulating and cutting vessels in narrow spaces [10].

LS system is composed of an energy generator and a hand instrument of different shapes and sizes for use in conventional or laparoscopic surgery. By melting the elastin and collagen of the vessel wall, LigaSure seals vessels, using bipolar pressure and thermal energy simultaneously. It has a feedback mechanism and ceases as soon as sealing is completed. It can seal vessels up to a diameter of 7 mm without proximal thrombus formation. It has been shown that a sealed vessel could resist pressures three times higher than normal systolic blood pressure [18].

TB is the world’s first and only advanced energy system that delivers two well-established forms of energy to a tissue simultaneously: ultrasonic energy for dissection and fast tissue-cutting capability and advanced bipolar energy for fast and secure haemostasis for vessels up to and including 7 mm in diameter. As the previous instruments the shears is available in different shapes and sizes for use in conventional or laparoscopic surgery [19].

### Statistics

Data were expressed as mean, unless otherwise specified. Statistical analysis was performed by SPSS 23th edition (SPSS©, Chicago, IL, USA), using two step cluster and t test paired functions. Results are expressed as mean ± standard deviation or percentage and significance was assigned with a *p* value < 0.05.

## Results

From January 2013 until June 2017, 75 patients (23 males, 52 females) were enrolled in the current study and were divided into three group of 25 patients each. The results for each group are reported in Table 1.

There was no significant statistical difference between groups regarding the relationship between male/female, right site/left site, the mean age, HT and the tumor size (*p* > 0.05).

Following the ASA criteria, 21 patients were classified ASA I, 38 patients ASA II and 16 patients ASA III.

To reach preoperative goals only two Conn’s disease patients needed supplementary potassium administration.

Conversion rate and mortality is nil in all groups despite of two intraoperative complication. In the G1 is reported a case of cava vein injury, laparoscopically sutured and treated with Floseal® Hemostatic Matrix (Baxter ZurichSwitzerland) and oxidized cellulose (Tabotamp Fibrillar Johnson & Johnson, NJ, US). In the G2 is reported a case of pleural cavity opening treated by suturing the diaphragm lesion.

Hypertensive crises were reported in 8 cases (2 PCC patients and 1 Cushing patient in G1, 3 PCC patients in G2, 1 PCC patients and 1 Cushing patient in G3), 5 at the induction, 3 during adenoma manipulation, while hypotensive crises were reported in 1 cases. No post-operative injuries, due to blood pressure, are reported thanks to prompt treatment. Only in one female PCC patient were reported a cardiac enzymes increasing in absence of myocardial infarction clinical manifestations.

**Fig. 1:**
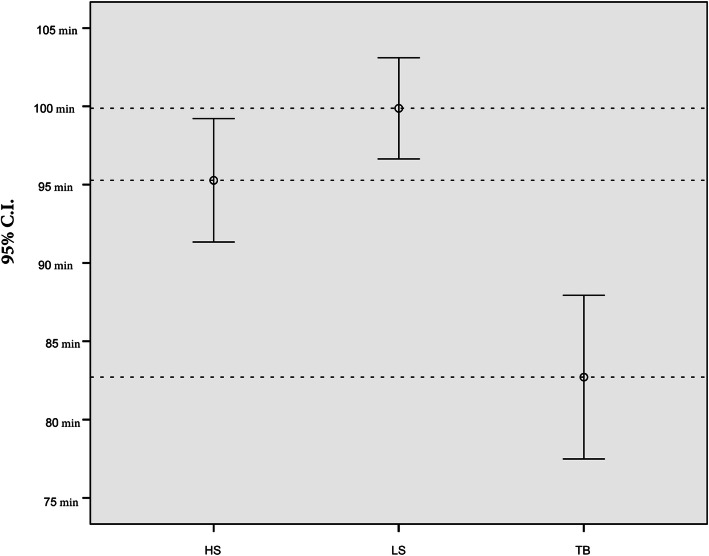
Operation Time error graph with 95% C.I

**Fig. 2:**
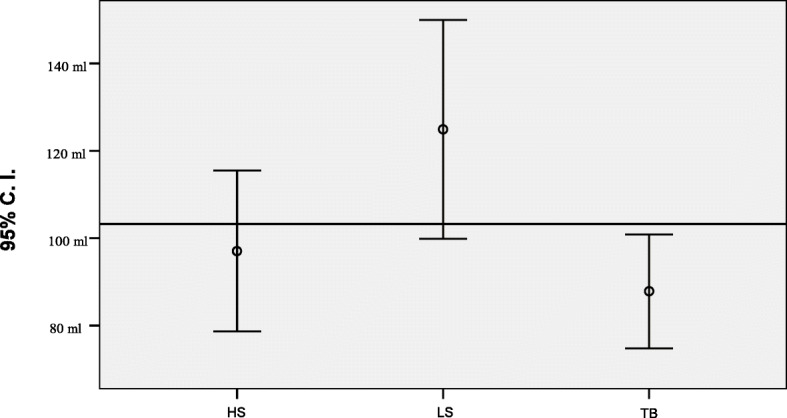
Intraoperative blood losses error graph with 95% C.I. Reference Line is the Overall Mean = 103,27

Significant statistical difference are detectable in OT and IBL. TB, compared respectively with LS and HS, is the fastest device (*p* < 0,001). The second faster device resulted HS, which meanly reduced the OT compared to LS (*p* = 0.048). IBL are reduced using TB (*p* < 0,001) and HS (*p* = 0.006) compared to LS, but between TB and HS there isn’t significant statistical difference (*p* = 0.178) Figs 1 and 2.

Functioning tumors were diagnosed in 47 patients (18 G1, 14 G2, 15 G3), 15 patients were affected by PCC (5 G1, 3 G2, 7 G3), 8 by Conn’s disease (2 G1, 5 G2, 1 G3), 5 by Cushing (3 G2, 2 G3).

Two patients affected by PCC presented RET proto-oncogene mutation, with a case of triple mutation (634, 640, 700) in the patient suffering from MEN 2A.

Surgery determined an hormonal serum levels normalization in 46 out of 47 diagnosed with functional adenoma (97.8%); only one patient affected by PCC disease showed a persistent postoperative hypertension and elevation of metanephrine levels, due to retrocaval adrenal tissue, requiring reoperation with posterior approach.

No postoperative mayor complication occurred. 30-day morbidity rate was 5.3% (4/75 patients) consisted of one case of abdominal wall hematoma, one case of pneumonia and two intra-abdominal collection spontaneously resolved.

## Discussion

Since the first successfully performed LA by Gagner in 1991 [3], the transperitoneal approach has became the most common therapeutic strategy for adrenal neoplasm, establishing itself nowadays like the gold standard technique [4–6, 20, 21].

Adrenal surgery is a field where precision and thoroughness are highly important. As in every surgical procedure, anatomical knowledge should always be associated with the more appropriate instrument. A comparison of new and advanced devices in a surgical reliable technique, yet not fully investigated, is not trivial.

Is easy to find in Literature data from the application of these new devices in thyroid surgery [12, 13, 22]. In fact, many papers have been published in last decade with this debated topic, but few data are available concerning the impact of these instruments on laparoscopic adrenalectomy.

Same Authors describe personal experiences with one device [23], other ones compare results between LS and HS or between these two devices with electric hook (14,15,18, 24) but no papers analyze and compare the use of these three technologies.

In thyroid surgery, a large British meta-analysis concluded that in a ranking scale, ultrasonic coagulation (HS) is in the first position in terms of reduced IBL and drain output, shortest OT and HT, followed by LS [12]. Unfortunately, in this analysis is not considered TB and it is targeted to a single specific tissue, making data not applicable to all anatomical sites.

Recently, an American group, tested the heat spread of LS, HS and TB in three different tissues concluding that all devices were similar with an heat lateral diffusion ≤2 mm [22] but, in Literature, is not possible to find a comparative study on the quality of coagulation or the haemostatic power of these three devices.

In the field of adrenal surgery, during 2008, an Italian group published a prospective study about the comparing of bipolar energy devices with ultrasonic ones (assisted by monopolar high-frequency) in laparoscopic adrenalectomy. The Authors demonstrated a significant difference in OT only for left-sided adrenalectomies and an overall decreased IBL in the bipolar energy devices group [24]. In 2010 Sartori et al., reported their experience of 46 patients underwent laparoscopic adrenalectomy using bipolar or ultrasound energy devices. The results could not demonstrate any differences in the surgical outcomes between the two groups and Authors concluded that hemostatic devices choice is up to surgeon’s preference [14]. In 2013 another Italian group analyzed, retrospectively, the difference between the use of bipolar, ultrasound and monopolar energy devices on 165 patients underwent laparoscopic adrenalectomy. Authors demonstrate that the use of advanced sealing devices is associated with a reduced OT, especially in left adrenalectomy, and with a better hemostasis when compared with monopolar energy devices. However, this study has some limitations represented by the retrospective analysis and the bias belonging from groups not well matched. [14, 15, 24]

To the best of our knowledge, the current study is the first comparing all commercially available advanced energy devices in laparoscopic adrenal surgery.

Following indication of most recent Literature, in the current study advanced energy devices are not compared with electric hook due to the evidence of a longer OT and HT and of an increasing IBL and drainage output associated to the use of the latter [15].

It must be highlighted that in the study groups there was no significant statistical difference regarding the relationship between male/female, right site/left site, the mean age and the side (*p* > 0.05). Therefore two step cluster and t test paired analysis were performed on our OT, IBL and HT results to evaluate the eventual superiority of an instrument over the others.

Is possible assert that TB is the best device to reduce OT with a significant statistical difference compared with LS and HS (*p* < 0.001). Probably, this data is consistent with the presence in a single device of both modality of advanced energy, conferring to the surgeon a greater speed of execution. Another significant statistical difference is observable between HS and LS (*p* = 0.048) probably due to the greater surgeon’s experience with the HS.

Regarding the IBL, between HS and TB no significant statistical difference were found (*p* = 0.17), nevertheless both reduced IBL in case of comparison with LS (*p* < 0.05).

No significant statistical difference were found in HT comparing each groups with the others but is very interesting to highlight the correlation between the use of advanced dissecting devices in laparoscopic adrenalectomy and shorter HT, leading to decreased costs, as evidenced by Valeri et al. in 2002 [25]. Unfortunately, this cost-benefit analysis, that demonstrate a considerable economic savings using HS, is not updated and not consider many economic and logistic variables changed in the last 15 years. In the current study, the Authors not performed a specific costs analysis of the use of the different energy devices. It would be interesting to analyse its economical implications in a future study.

The limitations of this article are all related to its retrospective nature. We are aware that the surgeon experience, his expertise or confidence whit one device may be a confounding factor affecting this study. However, all the procedures were performed by the same operator and the same surgical team. We therefore believe that the impact of this variable on the outcomes, if present, is minimal. Furthermore, is our opinion that the results of this study should not be considered true for other type of surgery but only in adrenal gland laparoscopic surgery.

## Conclusion

The current study was conceived to compare the three commercially available advanced energy device in LA. The Authors evaluated their effectiveness in terms of OT, IBL and HT. Analyzing the results, LAs carried out using TB appeared to show a statistically significant decrease in OT and IBL compared with LA performed using HS and LS, while HT was superimposable in all groups. According to our data, a responsible use of advanced energy devices can improve surgical outcomes guarantying a cost savings and patient’s satisfaction.

## Abbreviations

BP: Blood Pressure
HR: Heart Rate
HS: Harmonic Scalpel
LA: Laparoscopic Adrenalectomy
LS: Ligasure vessel sealing system
OT: Operation Time
PCC: Pheochromocytoma
SBP: Systolic Blood Pressure IBL Intraoperative blood losses
TB: Thunderbeat

## Authors' information

Gambardella Claudio and Mauriello Claudio are PhD student in Medical, Clinical and Sperimental Sciences at Univerity of Campania “Luigi Vanvitelli” – Naples, Italy.
